# Therapeutic expansion of JAK inhibitors in Chinese dermatological practice: beyond atopic dermatitis to novel autoimmune frontiers

**DOI:** 10.3389/fmed.2026.1752145

**Published:** 2026-08-26

**Authors:** Xinyan Lyu, Yixi Wang, Jingyi Yang, Yiwen Zhang, Wei Min, Na Du

**Affiliations:** 1 Department of Dermatology, Shanghai Fifth People’s Hospital of Fudan University, Shanghai, China; 2 Department of Dermatology, The First Affiliated Hospital of Soochow University, Suzhou, China; 3 Department of Dermatology, The First People’s Hospital of Kunshan, Kunshan, China

**Keywords:** alopecia areata, bullous pemphigoid, chronic spontaneous urticaria, dermatomyositis, JAK inhibitor, pemphigus, psoriasis, vitiligo

## Abstract

Janus kinase inhibitors (JAKi) have demonstrated clinical utility in managing atopic dermatitis (AD), yet their broader potential across immune-mediated dermatoses remains inadequately explored, particularly within China’s restrictive regulatory landscape for dermatologic indications. This review examines pathogenic mechanisms and emerging evidence supporting JAKi repositioning in seven dermatological conditions beyond AD: alopecia areata (AA), vitiligo, psoriasis, dermatomyositis (DM), pemphigus, bullous pemphigoid (BP), and chronic spontaneous urticaria (CSU)—with emphasis on therapeutic gaps and opportunities specific to Chinese practice. We delineate disease-specific JAK-STAT dysregulation, including IFN-γ-driven cytotoxicity in AA/vitiligo, IL-23/STAT3 activation in psoriasis, and type I interferon hyperactivation in DM. Key clinical findings from rigorous trials indicate that oral tofacitinib achieves PASI75 in 58% of psoriatic patients within 16 weeks and topical ruxolitinib induces facial repigmentation exceeding 75% in vitiligo. In contrast, while current evidence is substantially limited to isolated case reports, emerging data suggest that baricitinib may resolve steroid-refractory BP lesions within one month. Persistent limitations include reliance on small-scale studies for pemphigus and CSU, along with unresolved safety concerns regarding thrombosis risks in susceptible populations. Crucially, we identify therapeutic gaps in segmental vitiligo and juvenile DM where clinical data are absent. This synthesis advocates for randomized controlled trials to validate JAKi efficacy against biologics in high-burden diseases currently dependent on glucocorticoids.

## Introduction

### JAK-STAT pathway fundamentals and pharmacological rationale

The Janus kinase (JAK) signal transducer and activator of transcription (JAK-STAT) pathway functions as a critical cytokine transduction axis where ligand-receptor binding initiates phosphorylation cascades (1). Cytokine engagement induces transmembrane receptor dimerization, leading to reciprocal activation of associated JAK kinases of the JAK family (JAK1, JAK2, JAK3, TYK2) (2). These activated JAK kinases subsequently phosphorylate cytoplasmic signal transducers and activators of transcription (STATs), including STAT1, STAT2, STAT3, STAT4, STAT5A, STAT5B and STAT6 (3, 4). Phosphorylated STAT proteins form functional homodimers or heterodimers that translocate to the nucleus to modulate inflammatory gene transcription (5, 6). This phosphorylation cascade governs critical processes including cell differentiation, proliferation, and inflammatory responses, establishing JAK-STAT as a crucial regulator of immune homeostasis (7, 8).

JAK inhibitors (JAKi) are small molecules designed to competitively bind the ATP-binding site of JAK kinases, thereby blocking phosphorylation-dependent signal transduction (9). Their mechanism transcends single-cytokine targeting, enabling simultaneous disruption of multiple pathogenic axes (10). For instance, interferon-γ (IFN-γ) mediates the activation of JAK1/STAT1 signaling, leading to the activation of cytotoxic T helper 1 (Th1) immune responses, whereas IL-4 and IL-13 trigger JAK1-dependent STAT6 phosphorylation to promote Th2 cytokine polarization (11–13). While initially developed for hematologic malignancies (ruxolitinib in myelofibrosis), JAKi now hold FDA approvals for rheumatoid arthritis (tofacitinib), and ulcerative colitis (tofacitinib, upadacitinib). Additionally, JAKi baricitinib was granted FDA emergency authorization for hospitalized COVID-19 patients during the 2021 pandemic surge, underscoring the broad immunomodulatory utility of JAKi (14–18). Nevertheless, their broad-spectrum activity raises safety concerns, requiring rigorous benefit-risk evaluation for thrombosis, opportunistic infections, and potential malignancy before clinical deployment (19, 20).

### Chinese clinical context and unmet needs

In dermatology, JAKi have gathered most attention for atopic dermatitis (AD) globally (21). However, within China's regulatory landscape, approvals for JAKi in dermatology remain strictly limited. Currently, upadacitinib, abrocitinib, and ivarmacitinib are authorized for the treatment of moderate-to-severe AD. Although tofacitinib is approved in China, its approved indications in China are currently restricted to psoriatic arthritis. Nevertheless, several clinical studies suggest that tofacitinib demonstrates therapeutic efficacy against skin diseases, such as AD, and psoriasis, yet it has not received formal regulatory approval for these specific uses in China (22, 23).

**Figure 1:**
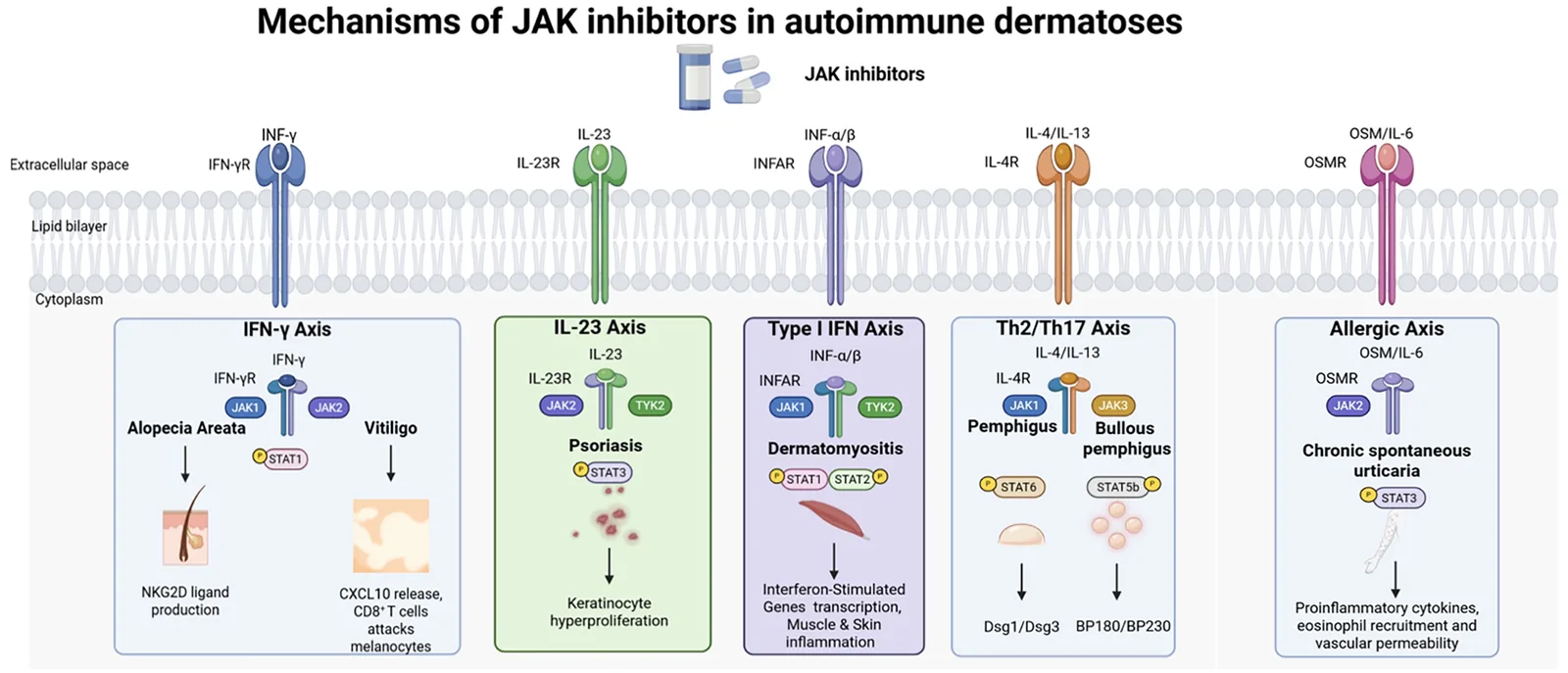
Mechanisms of JAK inhibitors in autoimmune dermatoses.

This limited approval ignores accumulating evidence which supports the effectiveness of JAKi in treating other immune-mediated skin diseases (Table 1) (24). Other immune-mediated dermatoses, besides AD, are struggling with restricted access to biologics and increasing dependence on steroids, especially for those with high disease burden and poor quality of life, such as alopecia areata (AA), vitiligo, psoriasis, dermatomyositis (DM), pemphigus, bullous pemphigoid (BP), and chronic spontaneous urticaria (CSU) (25–31). JAK-STAT dysregulation underpins the immunopathogenesis of several dermatoses (Figure 1) (32). For instance, IFN-γ-mediated JAK/STAT activation initiates cytotoxic T-cell destruction of hair follicles, leading to AA (33). Similarly, vitiligo pathogenesis involves IFN-γ-induced C-X-C motif chemokine ligand 10 (CXCL10) release through JAK-STAT signaling, recruiting autoreactive CD8+ T cells to attack melanocytes (34). Psoriasis relies on IL-23-activated JAK/STAT3 signaling in T cells, inducing IL-17 production that promotes keratinocyte hyperproliferation (35). In DM, type I interferons activate JAK/STAT pathway, amplifying inflammation in muscle and skin (36, 37). In pemphigus, the core mechanism involves T helper (Th)2 cells and IgG antibodies produced by B cells, which can be inhibited by JAK1/JAK3-STAT6 pathway (38). BP relies on JAK1/STAT3-STAT5b pathway, inducing the secretion of TNF-α, while CSU involves STAT3 hyperactivation in basophils and mast cells (39, 40). These mechanistic parallels suggest that JAK inhibition may achieve therapeutic efficacy comparable to that observed in AD.

**Table 1:** JAK inhibitors in global dermatology practice.

Regional approvals\JAK inhibitor	Tofacitinib	Baricitinib	Upadacitinib	Deucravacitinib	Ritlecitinib
Australia	Active psoriatic arthritis in adult patients	Moderate to severe atopic dermatitis in adult patients; severe alopecia areata in adult patients	Moderate to severe active psoriatic arthritis in adult patients; moderate to severe atopic dermatitis in adults and adolescents≥12 years old	Moderate to severe plaque psoriasis in adult patients	Severe alopecia areata in adult patients and adolescents ≥12 years old
Canada	Psoriatic arthritis	Severe alopecia areata in adult patients	Active psoriatic arthritis in adult patients; refractory moderate to severe atopic dermatitis in adult patients and adolescents ≥12 years old	Moderate to severe plaque psoriasis in adult patients	Severe alopecia areata in adult patients and adolescents ≥12 years old
China	Active psoriatic arthritis in adult patients	Severe alopecia areata in adult patients	Active psoriatic arthritis in adult patients; moderate to severe atopic dermatitis in adult patients and adolescents ≥12 years old	Moderate to severe plaque psoriasis in adult patients	Severe alopecia areata in adult patients and adolescents ≥12 years old
European union	Psoriatic arthritis in adult patients with red, scaly patches on the skin with inflammation of the joints; juvenile psoriatic arthritis	Moderate to severe atopic dermatitis in adult patients and children≥2 years old; severe alopecia areata in adult patients	Active psoriatic arthritis in adult patients; moderate to severe atopic dermatitis in adult patients and adolescents ≥12 years old	Moderate to severe plaque psoriasis in adult patients	Severe alopecia areata in adult patients and adolescents ≥12 years old
United states	Active psoriatic arthritis in adult patients and pediatric patients ≥2 years old	Severe alopecia areata in adult patients	Active psoriatic arthritis in adult patients and pediatric patients ≥2 years old who have had an inadequate response or intolerance to one or more TNF blockers; moderate to severe atopic dermatitis in adult patients and adolescents ≥12 years old	Moderate to severe plaque psoriasis in adult patients; active psoriatic arthritis in adult patients	Severe alopecia areata in adult patients and adolescents ≥12 years old

Based on the understanding of molecular mechanisms underpinning diverse immune-mediated dermatoses, as outlined in the preceding sections, this review specifically focuses on the therapeutic expansion of JAKi. While JAKi has established a prominent role in managing AD, compelling preclinical and emerging clinical evidence suggests significant potential beyond this single indication. The primary objective of this review is to synthesize translational and clinical evidence for JAKi utility in dermatoses beside AD, focusing on both established applications and emerging frontiers. We systematically assess the rationale and evidence supporting the clinical efficacy of JAKi for diseases including AA, psoriasis, DM, chronic urticaria, pemphigus, and BP. The emphasis is on combining molecular insights from these dermatoses with current discoveries from therapeutic trials and case reports, thereby investigating the off-label usage of JAKi. In this review, we aim to elucidate both the opportunities and current limitations of repositioning JAKi in dermatology. We hope to provide a clearer perspective on how JAKi might address unmet therapeutic needs in these challenging conditions.

### JAKi in China: approvals, innovation, and clinical translation

The adoption of JAKi in China is profoundly influenced by the National Medical Products Administration's restrictive approval framework and domestic pharmaceutical innovation. Among the three mechanistic classes—pan-JAK inhibitors targeting broad kinase activity, highly selective JAK1 inhibitors, and novel TYK2 allosteric inhibitors—only three agents currently hold dermatologic indications for moderate-to-severe atopic dermatitis. These include upadacitinib, abrocitinib, and China's first domestically developed JAK1 inhibitor ivarmacitinib. Off-label use persists for non-approved conditions such as alopecia areata and psoriasis due to limited formal indications.

China's strategic pipeline fills these gaps through targeted drug development. Ivarmacitinib, approved in 2025, achieved 66.1% EASI75 response vs. 21.6% in placebo (41). Current innovations include ivarmacitinib cream for mild-to-moderate atopic dermatitis in phase 3 trials, and envudeucitinib, a next-generation TYK2 inhibitor showing promising 78% PASI75 efficacy in moderate-to-severe plaque psoriasis (42, 43). These advances are guided by ***the 2025 Chinese Expert Consensus on JAK Inhibitors in Immune-Mediated Inflammatory Diseases***, which emphasizes comprehensive risk-benefit assessment for thromboembolism, major cardiovascular events, and malignancy prior to JAKi initiation. Surveillance parameters and frequency should undergo individualized adjustment based on clinical context, acknowledging differential adverse event profiles among JAKi subtypes.

Implementation challenges persist in real-world settings, with phase 3 clinical data revealing significant disease-dependent safety differentials. Ivarmacitinib demonstrated favorable tolerability in atopic dermatitis, where the 4 mg and 8 mg groups exhibited treatment-emergent adverse event rates comparable to placebo (69.0% and 66.1% vs. 64.9%, respectively), accompanied by less than 2.7% serious adverse event rates across all groups (41). However, rheumatoid arthritis trials showed notably higher adverse event incidence, with the 8 mg group reporting 90.5% treatment-emergent adverse events compared to 79.3% in placebo controls, demonstrating clinically important indication-specific variability (44). Beyond pharmacological factors, access disparities between urban and rural healthcare settings remain unresolved due to infrastructure limitations. To address these multidimensional barriers, mitigation strategies should integrate disease-specific safety protocols aligned with ***the 2025 Chinese Expert Consensus on JAK Inhibitors in Immune-Mediated Inflammatory Diseases*** while advancing tiered regional monitoring networks to systematically optimize pharmacovigilance across diverse populations.

## Method

To ensure a comprehensive synthesis of current evidence, this review employed a systematic approach to identify and evaluate literature on JAKi applications in dermatologic conditions beyond atopic dermatitis. Literature searches were conducted across PubMed, Embase, Cochrane Library, and Web of Science databases up to May, 2026. Search strategies combined Medical Subject Headings and free-text terms across two domains: Janus kinase inhibitors (e.g., “janus kinase inhibitor,” “JAKi,” “tofacitinib,” “baricitinib,” etc.), and target diseases (including “alopecia areata,” “vitiligo,” “psoriasis,” “dermatomyositis,” “pemphigus,” “bullous pemphigoid,” and “chronic spontaneous urticaria”). Boolean operators (AND/OR) optimized retrieval sensitivity.

Publications were assessed by two independent reviewers. Initial inclusion criteria prioritized on clinical studies reporting therapeutic outcomes in humans. Full-text review subsequently assessed articles meeting the following thresholds: randomized controlled trials and observational cohorts regardless of sample size; case series (≥3 patients); and mechanistically informative experimental studies. Case reports were included where they provided first evidence of efficacy in treatment-refractory disease or rare subtypes. Exclusion criteria removed non-English publications, animal-exclusive studies, and non-therapeutic investigations.

Due to the high heterogeneity among the studies (RCTs and case reports are mixed), a meta-analysis will not be conducted, but a qualitative synthesis will be carried out based on the evidence level. Two independent reviewers evaluated the methodological quality of the included studies using standard assessment tools tailored to the specific study designs. Randomized Controlled Trials (RCTs) were assessed using the Cochrane Risk of Bias (RoB 2.0) tool. Integrated analyses and cohort studies were evaluated via the Newcastle-Ottawa Scale (NOS). Single-arm clinical trials were appraised using the Methodological Index for Non-Randomized Studies (MINORS) scale. Case series and case reports were assessed using the respective Joanna Briggs Institute (JBI) Critical Appraisal Checklists. Discrepancies were resolved by consensus with a third reviewer.

### The application of JAK inhibitors in dermatoses

**Figure 2:**
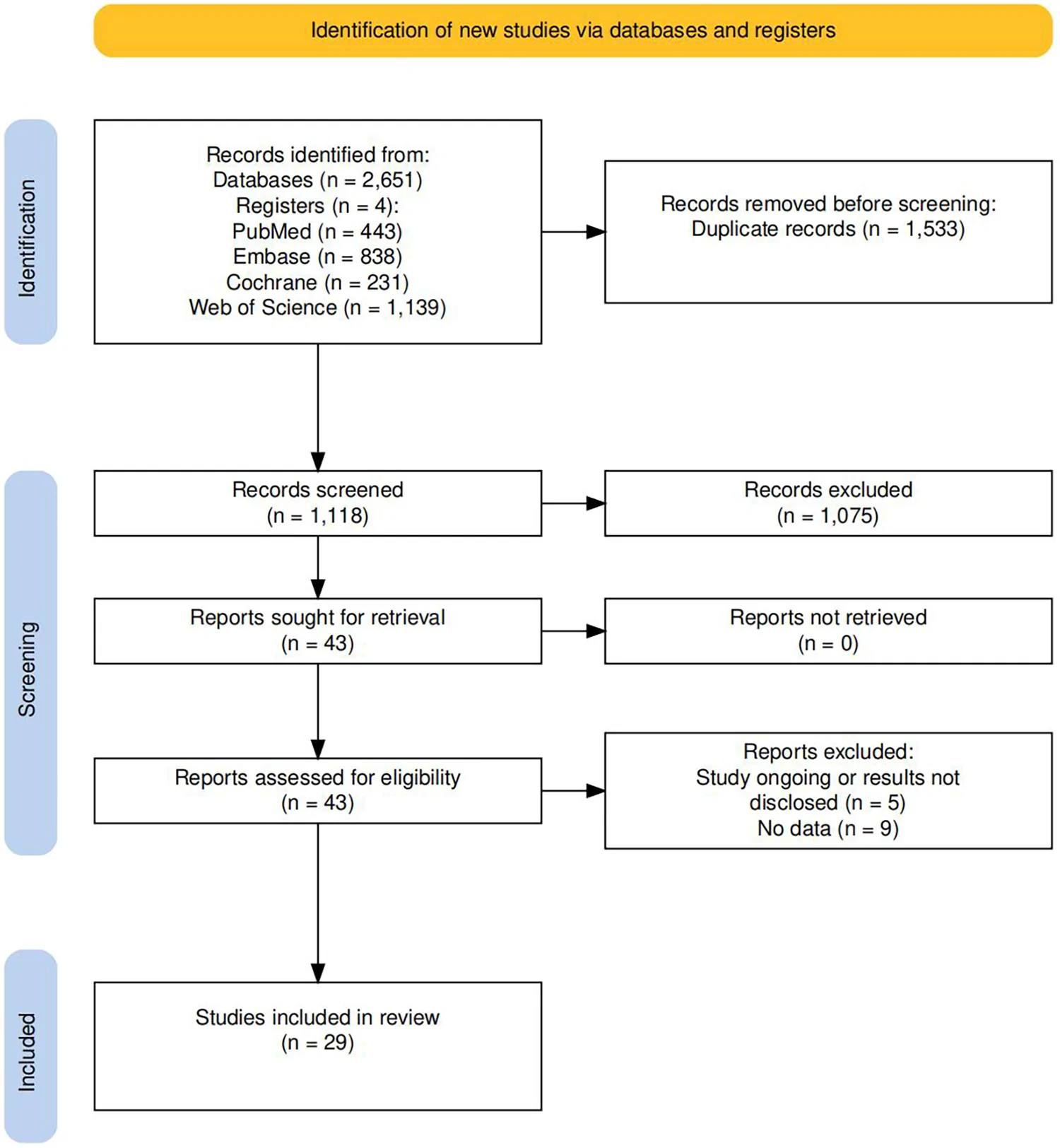
PRISMA 2020 flow diagram of the study.

A total of 29 studies were ultimately included in this systematic review. The detailed study selection process, from initial screening to final inclusion, is illustrated in the PRISMA 2020 flowchart (Figure 2). The included literature encompasses a broad spectrum of study designs, ranging from Phase 3 randomized controlled trials to exploratory case reports. To ensure rigorous evaluation, the summary of evidence levels and detailed quality assessments for all included studies are presented in Supplementary Table S1.

### Alopecia areata

Alopecia areata (AA) is an autoimmune disease posing a significant and evolving public health challenge. A recent analysis indicates that from 1990 to 2021, global incident cases surged from 20.43 to 30.89 million, despite a stable age-standardized incidence rate. The disease disproportionately affects females and individuals aged 30 to 34, with peak burdens in regions including North America and Southeast Asia. Crucially, AA is strongly linked to comorbidities such as depressive disorders and atopic dermatitis, transforming it into a complex systemic and psychological challenge. These trends highlight the urgent need for integrated, comorbidity-conscious management strategies in global and Chinese clinical practices (41).

The key pathogenesis of AA includes upregulation of MHC class I (MHC I), upregulation of UL16-binding protein 3 (ULBP3) molecules, or defect in natural killer (NK) cell activation, all of which leads to the defect in immune-privileged hair follicles (45). The NK cells or activated T cells then produced IFN-γ, which inturns induced the expression of MHC I, NKG2D and proinflammatory chemokines, forming a vicious loop of inflammation (46). The JAK/STAT3 signaling is the downstream of IL-6 which modulates NKG2D ligand levels, enabling the JAK/STAT pathway to participate in the pathogenesis of AA (47).

JAKi disrupt this AA-specific cascade by targeting IFN-γ-JAK/STAT signaling (48). Clinically, oral tofacitinib demonstrates significant efficacy. A landmark phase 2 trial (*N* = 66) reported 21% severity of alopecia tool (SALT) score improvement in patients after 3 months of 5 mg BID dosing, with 32% achieving 50% or greater improvement in SALT score (49). Beyond oral formulations, topical JAKi offer critical alternatives. Bayart et al. documented successful hair regrowth in 67% of pediatric patients using topical formulations, with improved safety profiles compared to systemic agents (50). This finding was corroborated by Putterman et al., where 8 of 11 patients showed measurable improvement with topical 2% tofacitinib (51). However, therapeutic decision-making requires consideration of regional accessibility. While topical agents reduce systemic risks, their limited follicular penetration in fibrotic scalps may explain treatment failures in chronic AA, as evidenced by the case report of ruxolitinib non-response in a 66-year-old female with long-standing disease (52).

Nevertheless, therapeutic limitations require careful consideration. Mackay-Wiggan et al. reported relapse in 33% (3/9) of responders within 12 weeks of ruxolitinib discontinuation (50), suggesting continuous JAK/STAT pathway dependency that necessitates protocolized tapering rather than abrupt cessation to prolong remission. Concurrently, hematologic monitoring remains imperative; both Mackay-Wiggan et al. and later large-scale analyses by King detected mild anemia in 1.3%–15% of JAKi-treated patients (53, 54), a manageable yet clinically relevant observation especially for populations with baseline cytopenias. Overall, JAKi represent promising therapeutic options for AA. Translating this promise into practice demands evidence-based stratification: for rapidly progressive alopecia totalis (AT) or alopecia universalis (AU), oral JAKi provide superior efficacy, while pediatric AA patients benefit from topical agents' favorable safety. Future randomized controlled trials (RCTs) should directly compare dose-optimization protocols against biologicals like IL-15 inhibitors, particularly addressing durability of response beyond initial treatment phases. The key clinical evidence supporting JAKi efficacy in AA, including dosage regimens, treatment responses, and safety profiles across pivotal studies, is consolidated in Table 2.

**Table 2:** Summary of key clinical studies on JAK inhibitors in alopecia areata.

References	Population	Intervention	Dosage	Duration	Outcomes	Adverse events
(46)	Adults with moderate to severe AA/AT/AU (*n* = 66)	Oral tofacitinib	5 mg BID	3 months	32% achieved ≥50% SALT improvement; median SALT score improvement: 21%	Infections; herpes zoster; headache; abdominal pain; acne; diarrhea; fatigue
(47)	Pediatric AA patients (*n* = 6)	Topical tofacitinib or ruxolitinib	Tofacitinib 1%–2% BID/QOD; Ruxolitinib 1%–2% BID (liposomal base)	3–18 months	4/6 patients had hair regrowth	Transient leukopenia; mild AST/ALT elevation
(48)	Pediatric AA/AT/AU patients (*n* = 11, ages 4–16)	Topical tofacitinib	Tofacitinib 2% BID/QD (liposomal base)	34.5 weeks	Average SALT improvement: 32.3%; 8/11 patients improved	Application-site irritation
(49)	66-year-old female with refractory AA	Topical ruxolitinib	Tofacitinib 0.6%, QD at Initial, BID after 2 months	3.5 months	No improvement	None
(50)	Moderate-to-severe AA patients (*n* = 12, mean age 44 years old)	Oral ruxolitinib	20 mg BID	3–6 months	9/12 achieved ≥50% hair regrowth	Mild infections; transient Hb drop
(51)	AA patients ≥12 years (*n* = 1,294)	Oral ritlecitinib	50 mg QD (± 4-week 200 mg load); 30/10 mg QD	624 days	≥55% patients achieved SALT≤20	Headache; COVID-19; nasopharyngitis; herpes zoster

AA: alopecia areata; AT: alopecia totalis; AU: alopecia universalis; SALT: severity of alopecia tool.

### Vitiligo

In vitiligo, melanocyte destruction is driven by autoreactive CD8+ T cells that infiltrate the epidermis through IFN-γ-CXCR3-CXCL10 chemokine axis (55–57). Compared with placebo, vitiligo patients had elevated IFN-γ (58). IFN-γ induced the secretion of CXCL10, recruiting autoreactive CD8+ T cells to epidermis, which relies on CXCR3 and CXCL10 to kill melanocytes (59). Meanwhile, IFN-γ receptor recruits JAKs to phosphorylate STAT1, which in turns promotes the transcription of genes induced by IFN-γ, including CXCL10 (59). This establishes a self-perpetuating inflammatory loop distinct from other autoimmune skin diseases.

JAKi primarily target this chemokine axis, suppressing T-cell recruitment rather than direct immune cell modulation. In a double-blinded trial, topical ruxolitinib 1.5% achieved F-VASI75 (≥75% facial repigmentation) in 52% of patients at week 52 while 45% patients achieved T-VASI50 (≥50% total repigmentation), which may attribute to regional variations in drug penetration or melanocyte reservoir accessibility (60). In a randomized phase 2b clinical trial, JAK3 (Janus kinase)/TEC (tyrosine kinase expressed in hepatocelluar carcinoma) inhibitor ritlecitinib demonstrated superior efficacy over placebo in patients with active non-segmental vitiligo (61). Clinical evidence regarding segmental vitiligo remains lacking.

Distinct safety profiles necessitate route-specific risk management. Hepatic enzyme elevations occur in 6.1% of patients receiving oral upadacitinib 11 mg (62), mandating baseline and quarterly liver function monitoring. In contrast, topical ruxolitinib maintains a favorable safety profile across patient subgroups with primarily localized adverse events (60). Future therapeutic development should focus on combination strategies with phototherapy to stimulate melanocyte migration from hair follicle reservoirs, particularly for recalcitrant non-facial lesions where monotherapy efficacy remains suboptimal. Critical efficacy metrics and safety outcomes from landmark trials of topical and oral JAKi in vitiligo are systematically summarized in Table 3.

**Table 3:** Summary of key clinical studies on JAK inhibitors in vitiligo.

References	Population	Intervention	Dosage	Duration	Outcomes	Adverse events
(57)	Adults with vitiligo (*n* = 157; mean age 48.3 years old)	Topical ruxolitinib	Ruxolitinib 1.5% BID/QD	Primary endpoint: 24 weeks; extension: 52 weeks (re-randomization of non-responders to higher doses)	Week 24: F-VASI50: 45% (1.5% BID) vs. 50% (1.5% QD); Week 52: F-VASI50: 58%, F-VASI75: 52%, F-VASI90: 33%; T-VASI50: 45%	Application site pruritus, acne
(58)	Adults with active nonsegmental vitiligo (*n* = 364)	Oral ritlecitinib	With loading dose: 200/50 mg vs. 100/50 mg for 4 weeks, followed by maintenance dosing of 50 mg daily for 20 weeks; without loading dose: 50 mg, 30 mg, 10 mg daily	Dose-ranging: 24 weeks; extension: 24 weeks (non-responders to ritlecitinib 200/50 mg)	Week 24: F-VASI % CFB: Significant for 50 mg ± LD & 30 mg vs. placebo; F-VASI75 response: 7.7%–12.1% for 50 mg ± LD	Nasopharyngitis, upper respiratory tract infection, headache
(59)	Adults with extensive non-segmental vitiligo (*n* = 185)	Oral upadacitinib	6 mg/11 mg/22 mg QD	Primary endpoint: 24 weeks; extension: 52 weeks	Week 52: F-VASI75: UPA11 (63.2%), UPA22 (37.9%); T-VASI50: UPA11 (39.5%), UPA22 (41.4%)	COVID-19, headache, acne, fatigue, hepatic disorder, anaemia

F-VASI75: ≥75% facial repigmentation; T-VASI50 ≥50% total repigmentation; CFB: change from baseline; LD: loading dose; UPA11: upadacitinib 11 mg/day; UPA22: upadacitinib 22 mg/day.

### Psoriasis

The underlying pathogenesis of psoriasis, a chronic inflammatory skin disease, is dysregulated immune responses that converge on the IL-23/Th17 axis (60). IL-23 promotes the expansion of Th17 lymphocytes, which secrete effector cytokines including IL-17 and IL-22 (63). These cytokines, particularly IL-17A and IL-22, bind to keratinocyte surface receptors respectively while modulate STAT3 activation, initiating intracellular JAK-STAT signaling cascades (64, 65). Sustained STAT3 signaling induces hyperproliferation of keratinocytes, inhibits normal differentiation, and amplifies inflammation by upregulating chemokines like CXCL1 and CCL20 (66, 67). Genetic evidence supports this mechanism since polymorphisms in the STAT3 gene correlate with psoriasis susceptibility (68).

JAKi disrupt this cascade by blocking IL-23 receptor signaling through inhibition of JAK1/TYK2 kinases, thereby suppressing Th17 differentiation, as well as inhibiting downstream STAT3 phosphorylation which mitigates transcription of pro-inflammatory mediators such as β-defensin 3 while restoring fibroblast homeostatic function (69, 70). Clinically, this dual mechanism translates to superior efficacy in anatomically resistant lesions. In a phase 3 randomized non-inferiority trial, oral tofacitinib 10 mg BID achieved 50% improvement in Nail Psoriasis Severity Index (NAPSI50) in 44.2% patients vs. 12.0% with placebo (*p* < 0.001), addressing a longstanding therapeutic gap in nail psoriasis management (71).

In a phase 3 randomized non-inferiority trial, oral tofacitinib 5 mg BID attained 75% improvement in Psoriasis Area and Severity Index (PASI75) in 39.5% patients vs. 2.7% placebo (*p* < 0.0001) at week 12 (72). Although only one adjudicated cardiovascular event occurred in the tofacitinib group vs. none in placebo, broad JAK1–3 inhibition carries class-level warnings for thrombosis (72). Comparatively, a phase III trial of selective TYK2 inhibitor, deucravacitinib, has achieved PASI75 in 58.4% patients at week 16, significantly outperforming placebo (12.7%) and apremilast (35.1%) without JAK-associated hematologic risks (73). This establishes TYK2 inhibition as a strategic alternative for patients with cardiovascular comorbidities.

While nail-specific efficacy demonstrates JAKi’s potential in anatomically complex lesions, responses in other difficult-to-treat areas like scalp and palmoplantar sites remain limited in pivotal studies. Future trials should prioritize anatomical region-stratified efficacy analysis for phenotype-specific optimization, as well as exploring synergistic strategies for patients who respond poorly to monotherapy. Efficacy and safety data of JAKi in pivotal psoriasis trials is synthesized in Table 4.

**Table 4:** Summary of key clinical studies on JAK inhibitors in psoriasis.

References	Population	Intervention	Dosage	Duration	Outcomes	Adverse events
(69)	Adults with moderate to severe plaque psoriasis and nail involvement (*n* = 1,196)	Oral tofacitinib vs. placebo	Tofacitinib 5/10 mg BID; placebo: BID	16 weeks	NAPSI50: tofacitinib 10 mg BID (44.2%) vs. placebo (12.0%); NAPSI75: tofacitinib 10 mg BID (28.1%) vs. placebo (6.8%)	Safety consistent with known JAK inhibitor profiles
(70)	Adults with moderate to severe chronic plaque psoriasis (*n* = 1,106)	Oral tofacitinib vs. subcutaneous etanercept vs. placebo	Tofacitinib 5 mg BID; etanercept 50 mg twice weekly; placebo: BID	12 weeks	PASI50: tofacitinib 5 mg BID (65.7%) vs. placebo (2.6%); PASI75: tofacitinib 5 mg BID (39.5%) vs. placebo (2.7%)	Nasopharyngitis, upper respiratory tract infection, cardiac events
(71)	Adults with moderate to severe plaque psoriasis (*n* = 666)	Oral deucravacitinib vs. oral apremilast vs. placebo	Deucravacitinib: 6 mg once daily; apremilast: 30 mg twice daily; placebo: BID	16 weeks	PASI75: deucravacitinib (58.4%) vs. apremilast (35.1%) vs. placebo (12.7%)	Nasopharyngitis, upper respiratory tract infection

NAPSI, Nail Psoriasis Severity Index; NAPSI50, 50% reduction from baseline in NAPSI score; NAPSI75, 75% reduction from baseline in NAPSI score.

### Dermatomyositis

Dermatomyositis (DM) is an idiopathic inflammatory myopathy (IIM) correlated with genetic risk factors, environmental triggers, and dysregulated immunopathogenesis (74). Disruption of intracellular antigens, comprising Mi-2, TIF1γ, and MDA5, initiates the type I interferon (IFN) signaling (75). IFNα and IFNβ activate JAK1, TYK2, STAT1, and STAT2 to start the transcription of IFN-stimulated genes (ISGs), while upregulating autoantigens, forming a vicious loop (76). These myositis-specific autoantibodies trigger perivascular inflammation, endothelial damage, and complement deposition in dermal and muscle micro vessels, leading to ischemia and perifascicular atrophy (77).

JAKi target this pathway by blocking IFN receptor signaling, thereby suppressing pathogenic ISG expression (78, 79). Clinical evidence supports their efficacy in refractory DM: An open-label pilot study reveals that at week 12, tofacitinib (11 mg extended-release daily) significantly reduced Cutaneous Dermatomyositis Disease Area and Severity Index (CDASI) scores (*P* = 0.0005), as well as normalizing STAT1 signaling in skin biopsies (80). In addition, serum chemokine levels of CXCL9/CXCL10 showed a statistically significant decrease from baseline (80).

Significantly, treatment response heterogeneity underscores the need for endotype-driven strategies. In anti-MDA5 antibody-positive interstitial lung disease (ILD), tofacitinib significantly improved radiological outcomes and survival; however, baseline hyperferritinemia (>1,000 ng/mL) identified patients at high risk of lethal respiratory failure, with a 40% mortality rate observed in refractory cases despite combined immunosuppressive therapy (81, 82). Similarly, baricitinib (4–8 mg/day) improved CDASI and muscle strength in juvenile DM patients over 24 weeks, highlighting its value as a steroid-sparing agent (83).

Meanwhile, safety concerns remain when it comes to the administration of JAKi in patients with DM. For instance, risks of herpes zoster reactivation and infection have been reported in several case reports, requiring vigilant monitoring during treatment (84, 85). A case report highlighted that during the treating process, one patient showed pancytopenia, while two patients had shock of an unknown cause and intramuscular bleeding, whereas these adverse events correlated with JAKi remains unknown (82). To date, clinical evidence for JAKi in DM remains limited despite emerging case reports, compelling more studies for definitive therapeutic validation.

Traditionally, JAK inhibitors have been positioned primarily as salvage options for refractory DM. However, this therapeutic paradigm is rapidly evolving. Emerging evidence specifically highlights upadacitinib's potential not merely as a rescue agent, but as a highly effective first-line therapy for controlling recalcitrant skin manifestations in DM (84). This evolving perspective suggests that selective JAK inhibition could be integrated much earlier into the clinical treatment algorithm for cutaneous DM.

Collectively, these findings position JAKi as promising agents for refractory DM, though larger prospective studies remain imperative to establish long-term efficacy and optimal monitoring protocols. Future research should prioritize on long-term safety surveillance for infection and hematologic events, and comparative studies differentiating JAKi efficacy across distinct autoantibody profiles. Clinical outcomes and safety observations from studies of JAKi in refractory dermatomyositis, including high-risk anti-MDA5+ subgroups, are detailed in Table 5.

**Table 5:** Summary of key clinical studies on JAK inhibitors in dermatomyositis.

References	Population	Intervention	Dosage	Duration	Outcomes	Adverse events
(78)	Adults with refractory dermatomyositis (*n* = 10)	Oral tofacitinib	11 mg QD	12 weeks	50% achieved moderate improvement, 50% achieved minimal improvement; CDASI: 28 at initial, 9.5 after treatment; serum CXCL9 (*p* = 0.049), and CXCL10 (*p* = 0.013) siginificantly decreased	Urinary tract infection
(79)	Adults with early-stage anti-MDA5 + amyopathic dermatomyositis-associated intestinal lung disease (*n* = 18)	Add-on oral tofacitinib to background therapy	5 mg BID	6 months	Tofacitinib group achieved 100% 6-month survival vs. 78% in historical controls	Hyperlipidemia, leukopenia, acne
(80)	Refractory rapidly progressive interstitial lung disease associated with anti-MDA5 + dermatomyositis (*n* = 5)	Add-on oral tofacitinib to background therapy	5 mg BID	4–18 weeks	Tofacitinib group achieved 60% survival vs. 0% in matched historical controls; baseline hyperferritinemia (>1,000 ng/mL) identified patients at high risk of lethal respiratory failure	CMV reactivation; herpes zoster; bacterial/fungal infections
(81)	Adolescents with refractory juvenile dermatomyositis (*n* = 4)	Oral baricitinib	2–4 mg BID	24 weeks	All patients improved CDASI and muscle strength	Upper respiratory infection

CDASI, cutaneous dermatomyositis disease area and severity index; CMV, cytomegalovirus.

### Pemphigus

Pemphigus encompasses life-threatening autoimmune blistering diseases mediated by pathogenic IgG autoantibodies targeting desmosomal adhesion proteins, primarily desmoglein (Dsg) 1 and Dsg3 (86). This autoimmune targeting destroys intercellular adhesion complexes, triggering and subsequent mucocutaneous blistering (87). Mounting mechanistic evidence underscores the pivotal involvement of the JAK/STAT signaling cascade in disease immunopathogenesis. Pemphigus pathogenesis is critically mediated by cytokine networks reliant on Janus kinase signal transduction (88). Key mediators including IL-6, IL-4, IL-17, IL-21, and IFN-γ require JAK activation to propagate autoimmune inflammation (89). Furthermore, T helper 2 polarized cytokines such as IL-4 and IL-13 activate JAK1 and JAK3 dependent STAT6 signaling pathways (90). This cascade stimulates B lymphocyte differentiation toward pathogenic immunoglobulin G4 autoantibody production (91). Simultaneously, increased phosphorylation of JAK1 and JAK2 activates downstream pathways in pemphigus vulgaris, contributing to keratinocyte apoptosis and loss of intercellular adhesion (92). Histopathology of pemphigus vulgaris lesions further corroborate this mechanism, demonstrating significantly elevated phosphorylated JAK1 and JAK2 in keratinocytes compared to healthy skin, with a tendency toward higher expression in clinically active lesions (92).

While JAKi show mechanistic plausibility for pemphigus, current clinical evidence necessitates cautious interpretation due to exclusive reliance on case-level data. Clinically, targeted JAK inhibition demonstrates therapeutic potential in recalcitrant pemphigus variants. In mucous membrane pemphigoid (MMP), JAKi demonstrated rapid efficacy: baricitinib (4 mg/day) achieved significant reduction in conjunctival inflammation within 2 months in a rituximab-refractory case, while tofacitinib (11 mg/day) induced durable remission (>12 months) in patients failing intravenous immunoglobulin, mycophenolate mofetil, and rituximab therapy (93, 94). For dermatitis herpetiformis and epidermolysis bullosa, tofacitinib (10 mg/day) abolished new blister formation in dapsone-intolerant dermatitis herpetiformis after 7 months, while both tofacitinib and baricitinib alleviated debilitating pruritus within 4 weeks in epidermolysis bullosa pruriginosa resistant to topical steroids (95–97). Recently, Hren et al. reported that udpadacitinib alleviated refractory mucosal erosions in a case of pemphigus vulgaris (98).

These exploratory evidence from case reports constrain JAKi exclusively to salvage therapy for multidrug-refractory pemphigus subtypes. Three fundamental limitations warrant emphasis in clinical consideration. First, evidence originates almost entirely from off-label use after failure of standard regimens, notably lacking controlled comparisons with first-line therapies. Second, durability beyond 12 months remains minimally documented aside from one exceptional report of 24-month remission; longer-term efficacy validation represents an unmet need. Finally, insufficient data exist regarding infectious complications in this chronically immunosuppressed population. Future investigations should establish disease-specific registries to track relapse patterns and adverse event profiles, alongside efficacy trials against established treatments. Pending robust evidence generation, JAKi must be reserved as a monitored rescue intervention. Therapeutic outcomes and adverse events from published reports of JAKi use in pemphigus spectrum disorders are compiled in Table 6.

**Table 6:** Summary of key clinical studies on JAK inhibitors in pemphigus.

References	Population	Intervention	Dosage	Duration	Outcomes	Adverse events
(92)	43-year-old male patient with severe refractory mucous membrane pemphigoid	Add-on oral baricitinib to background therapy	4 mg QD	4 months	Stabilization of disease progression (first time in 8 years); reduced conjunctival hyperemia/corneal neovascularization	Transient neutropenia
(93)	Refractory ocular mucous membrane pemphigoid (79-year-old female and 70-year-old male)	Add-on oral tofacitinib to background therapy	11 mg QD	Case 1: 8 months; case 2: 8 weeks	Reduced conjunctival injection by 8 weeks	None
(94)	76-year-old male patient with refractory dermatitis herpetiformis	Add-on tofacitinib to ongoing monthly IVIG therapy	5 mg BID	7 months	Significant pruritus reduction and near-complete clearance by 1 month; sustained at 7 months	None
(95)	40-year-old male patient with pidermolysis bullosa pruriginosa	Oral baricitinib	2 mg QD	24 weeks	Significant pruritus improvement at day 2; papules and nodules began to resolve 2 weeks later without recurrence of blisters	None
(96)	26-year-old female with epidermolysis bullosa pruriginosa	Oral tofacitinib	5 mg BID	4 weeks	VAS decreased from 9 to 6, EBDASI reduced from19 to 15; local lichenoid papules decreased, and the original plaques became flat	None
(97)	64-year-old male with refractory mucosal pemphigus vulgaris	Add-on oral udpadacitinib to ongoing rituximab and IVIG	15 mg QD	6 months	Heal of oral lesions and no emerging lesions by 2 months, heal of all previous lesions by 6 months	None

IVIG, intravenous immunoglobulins; EBDASI, Epidermolysis Bullosa Disease Activity and Scarring Index; VAS, visual analogue scale.

### Bullous pemphigoid

Bullous pemphigoid (BP) is an autoimmune blistering disorder characterized by autoantibodies targeting BP180 and BP230 antigens at the dermal-epidermal junction (99). The JAK-STAT pathway contributes to BP pathogenesis by transducing signals from key cytokines (100). Elevated Th2 cytokines (IL-4, IL-5, IL-13) and Th17 cytokines (IL-17, IL-23) in blister fluid and serum activate JAK/STAT pathway, promoting eosinophil recruitment, neutrophil protease release, and autoantibody production (88). JAKi may disrupt this cascade by blocking downstream phosphorylation of STAT proteins, reducing inflammation and blister formation (101).

Nevertheless, the transition from pathophysiology to clinical validation faces significant evidence constraints. To date, reported therapeutic outcomes derive entirely from isolated rescue scenarios following conventional therapy failure. A case report described rapid remission with baricitinib (4 mg/day) in a glucocorticoid-resistant BP patient, with reduced eosinophil counts and anti-BP180 titers within 4 weeks (102). Similarly, tofacitinib (10 mg/day) achieved complete remission in patients who were resistant to biologics like dupilumab, evidenced by decreased pruritus scores and lesion amelioration (103). Conversely, trials targeting single cytokines show variable results. Mepolizumab (anti-IL-5) failed to improve relapse rates despite reducing eosinophils, while ustekinumab (anti-IL-12/23) occasionally triggered new-onset BP in psoriasis patients (104, 105). Ongoing trials comparing JAKi against standard biologics are critical. The ongoing phase III dupilumab trial (NCT04206553) aimed to evaluate whether dupilumab demonstrated superior remission rates over glucocorticoids, but similar head-to-head data for JAKi remain absent (106). Given the sole reliance on uncontrolled case evidence without longitudinal safety assessment, we assert that JAKi should be reserved strictly for empirical salvage therapy in refractory BP. Consequently, future research should elucidate the therapeutic profile of pan-JAK inhibitors against cytokine-specific agents in distinct BP phenotypes, with particular emphasis on establishing safety thresholds for elderly patients with comorbidities, given their heightened vulnerability to treatment-related complications. Table 7 presents clinical evidence for JAKi in bullous pemphigoid, including combination therapies and comparative data.

**Table 7:** Summary of key clinical studies on JAK inhibitors in bullous pemphigoid.

References	Population	Intervention	Dosage	Duration	Outcomes	Adverse events
(101)	83-year-old male with concurrent plaque psoriasis and bullous pemphigoid	Oral baricitinib	4 mg QD	24 weeks	BP lesions and psoriasis plaques achieved complete remission; pruritus attenuated; anti-BP180 and eosinophil counts significantly decreased	None
(102)	73-year-old male with refractory bullous pemphigoid	Oral tofacitinib combined wth low-dose glucocorticoids	5 mg BID	28 weeks	BPDAI: from 46.5 to 0; Anti-BP180: from 141.29 IU/mL to 31.02 IU/mL; Anti-BP-230: from 99.55 IU/mL to 43.15 IU/mL; histologic resolution	None
(103)	BP patients (*n* = 30)	Intravenous mepolizumab + OCS vs. placebo + OCS	Mepolizumab 750 mg IV every 4 weeks	12 weeks	Relapse-free rates were similar between groups; blood eosinophils significantly reduced in mepolizumab group	No drug-related adverse events
(105)	Adults with moderate to severe BP (*n* = 98)	Intravenous dupilumab + OCS vs. placebo + OCS	Dupilumab 600 mg subcutaneous initially; followed by dupilumab 300 mg subcutaneous every 2 weeks	52 weeks	Ongoing study	Ongoing study

BPDAI, Bullous Pemphigoid Disease Area Index; OCS, oral corticosteroids; IV, intravenous.

### Chronic spontaneous urticaria

Chronic spontaneous urticaria (CSU) is characterized by recurrent wheals and angioedema persisting beyond six weeks, driven largely by dysregulated mast cell activation and inflammatory cytokine cascades (107). The JAK-STAT pathway plays a pivotal role in CSU pathogenesis, as upregulated OSMR expression activates JAK2/STAT3 signaling, promoting proinflammatory cytokines such as IL-6, IL-9, and IL-10, which contribute to eosinophil recruitment and vascular permeability (39). Bioinformatics analyses of lesional skin further confirm enrichment of JAK-STAT, TNF, and NF-κB pathways, with IL-6 identified as a hub gene correlated with immune cell infiltration (108). JAKi disrupt this cascade by attenuating mast cell degranulation and downstream inflammatory mediator production (109, 110).

Clinically, JAK inhibition shows promise in refractory CSU. A phase 2 trial demonstrated that oral JAK1 inhibitor povorcitinib provided clinically significant efficacy in 136 antihistamine-refractory CSU patients (111). At week 12, patients who achieved povorcitinib (75 mg/day) achieved superior weekly Urticaria Activity Score (UAS7) reduction over placebo while maintaining a favorable safety profile (111). In our previous study, we reported a case series of five omalizumab-resistant patients (112). In those patients who failed traditional treatments and novel agents, abrocitinib (100 mg/day) achieved significant reductions in UAS7, with all patients attaining complete remission within 2–8 months without major adverse events (112). Nevertheless, the uncontrolled design and small sample sizes of such investigations served as exploratory evidence, imposing significant constraints on treatment-related conclusions. Similarly, murine models demonstrate that OSMR silencing suppresses JAK2/STAT3 activation, mitigating histamine release and eosinophil infiltration (110). However, findings from murine models remain exploratory and are insufficient to overcome the limited evidence from human studies.

Nonetheless, JAK inhibitor efficacy in CSU requires validation through large-scale randomized controlled trials, as current evidence derives primarily from small case series. Furthermore, JAK inhibition may incompletely target inflammatory pathways such as TLR4/NF-κB signaling, which bioinformatic analyses has already identified as co-activation hubs in CSU lesional skin. Table 8 summarizes the limited but promising clinical data on JAKi in refractory CSU, including translational evidence from murine models.

**Table 8:** Summary of key clinical studies on JAK inhibitors in chronic spontaneous urticaria.

References	Population	Intervention	Dosage	Duration	Outcomes	Adverse events
(109)	Human: 148 CSU patients vs. 148 normal controls Murine: 72 CSU-modeled mice vs. 8 controls	Human: autologous serum skin test, histamine release test, RT-qPCR Murine: intraperitoneal IL-9/IL-10/AG490 (JAK/STAT inhibitor)	Human: none Murine: IL-9/IL-10 plasmids; AG490 20 μmol/L	/	Human: In CSU patients, histamine release rates were elevated; expression of IL-9, IL-10, STAT3, and JAK was increased while the expression of INF-γ was decreased in the skin tissues Murine: IL-9/IL-10 promoted CSU via JAK/STAT pathway; inhibition of JAK/STAT mitigated histamine release and eosinophil infiltration	/
(110)	Adults with H_1_-antihistamine-refractory chronic spontaneous urticaria (*n* = 136)	Oral povorcitinib	Povorcitinib: 15/45/75 mg QD; placebo: QD	12 weeks	Povorcitinib (75 mg/QD) group achieved superior UAS7 least squares mean (LSM) change vs. placebo (*P* = 0.047); Povorcitinib (75 mg/QD) group achieved 62.9% disease control (UAS7 ≤ 6) and 42.9% complete response (UAS7 = 0)	None
(111)	Adults with refractory CSU (*n* = 6)	Oral abrocitinib	100 mg QD	2–8 months	Mean UAS7 reduced from 32.2 to 5.0; 5/6 patients achieved complete remission	Transient ALT elevation

### Comparative analysis of therapeutic responses across dermatoses

The heterogeneous efficacy of JAKi across dermatologic conditions emerges from disease-specific JAK-STAT pathway engagement, tissue barrier properties, and accessibility of target cell populations. AA and vitiligo exhibit high responsiveness to JAKi due to dominant IFN-γ-driven JAK/STAT signaling, which directly promotes cytotoxic CD8+ T-cell destruction of melanocytes, and NKG2D-mediated inflammation of hair follicles. This mechanistic vulnerability is clinically evidenced by ≥55% achieving SALT≤20 in AA with ritlecitinib and 52% achieving F-VASI75 in vitiligo with topical ruxolitinib. For psoriasis, inhibition of IL-23-induced JAK/STAT3 activation in keratinocytes underlies the efficacy of both pan-JAK inhibitors (tofacitinib achieving NAPSI50 in 44.2% for nail psoriasis) and selective agents (deucravacitinib achieving PASI75 in 58.4%), though tissue-specific penetration challenges persist in scalp and palmoplantar regions. DM shows JAKi sensitivity in type I interferon-high subtypes, but clinical utility is counterbalanced by elevated infection risks. Crucially, pemphigus and BP demonstrate inconsistent responses despite Th2/Th17 cytokine involvement, with evidence restricted to salvage case reports. Poor efficacy generalizability here likely stems from redundant non-JAK pathways and persistent autoantibody production in pemphigus.

Differences in topical drug delivery critically influence JAKi efficacy. Facial vitiligo shows superior repigmentation due to enhanced skin permeability, whereas structural barriers in AA, particularly scalp fibrosis, restrict drug penetration into hair follicles. These findings confirm that JAK inhibitors are context-dependent therapeutics governed by dominant signaling pathways and anatomical constraints.

## Conclusion

### Therapeutic achievements

**Figure 3:**
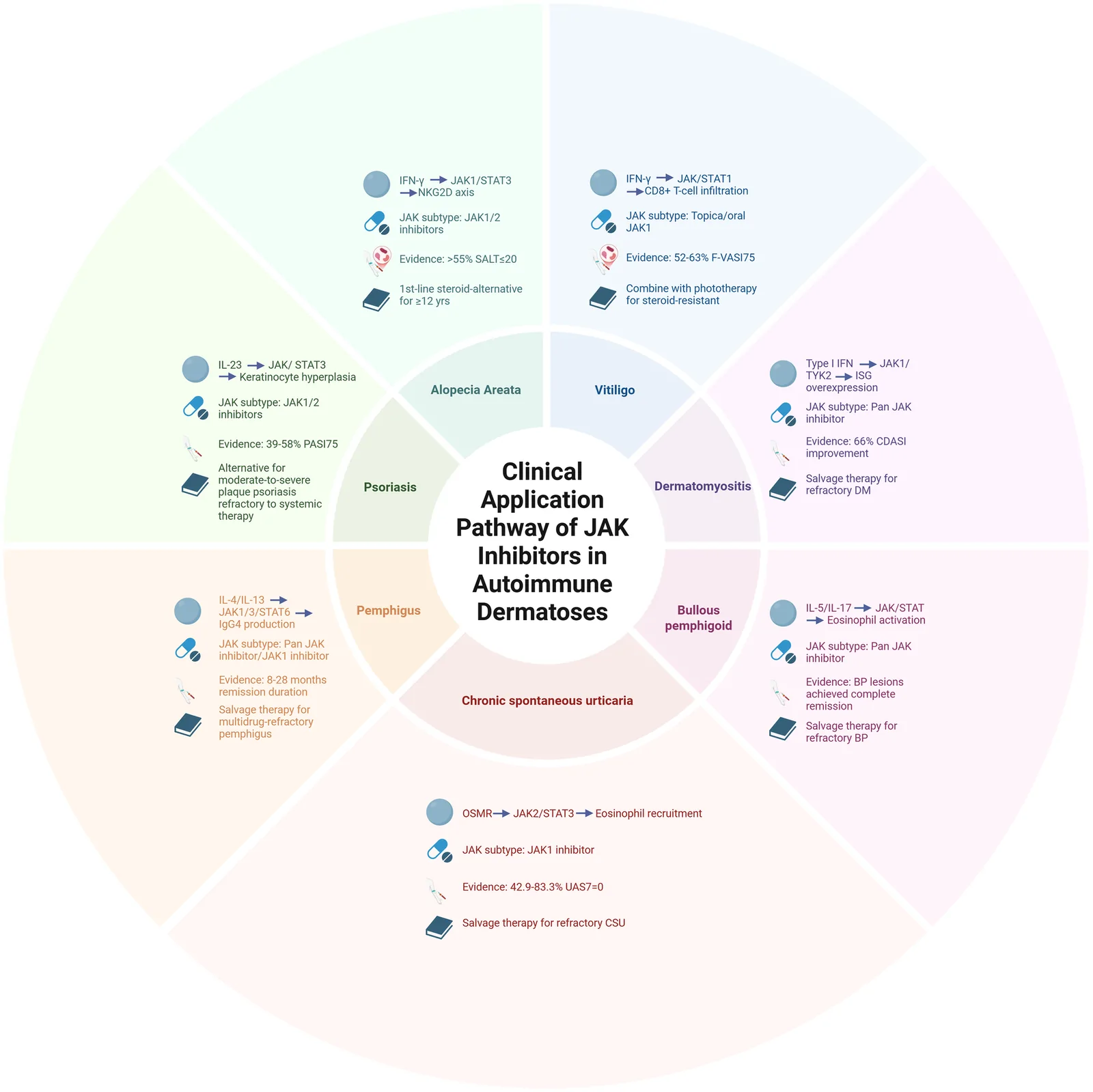
Clinical application pathway of JAK inhibitors in autoimmune dermatoses.

This systematic review consolidates translational and clinical evidence supporting JAKi repositioning across seven immune-mediated dermatoses beyond AD (Figure 3). As systematically compared in Table 9, the pathogenic drivers, clinical evidence levels, key efficacy metrics, and therapeutic positioning of JAKi vary substantially among distinct autoimmune dermatoses, ranging from IFN-γ-driven cytotoxicity in AA and vitiligo to IL-23/STAT3 hyperactivation in psoriasis and type I interferon amplification in DM. Clinically, JAKi exhibit compelling efficacy in recalcitrant cases: achieving PASI75 in 58% of psoriatic patients, inducing >75% facial repigmentation in vitiligo, and resolving steroid-refractory BP lesions within weeks.

**Table 9:** Comparative therapeutic profiles of JAKi across autoimmune dermatoses.

Disease	Core pathogenic pathways	Clinical evidence level (GRADE)	Key efficacy metrics	Position in therapy
Alopecia Areata	IFN-γ → JAK1/STAT3 → NKG2D-mediated inflammation	High Quality	≥ 55% patients achieved SALT ≤ 20	Severe alopecia areata in adult patients
Vitiligo	IFN-γ → JAK/STAT1 → CD8+ T-cell infiltration	High Quality	F-VASI75: 52%–63%	Alternative to traditional therapy
Psoriasis	IL-23 → JAK/STAT3 → Keratinocyte hyperplasia	High Quality	PASI75: 39%–58%	Alternative to traditional therapy
Dermatomyositis	Type I IFN → JAK1/TYK2 → ISG overexpression	Moderate Quality	CDASI improvement: 66%	Salvage for refractory dermatomyositis
Pemphigus	IL-4/IL-13 → JAK1/3/STAT6 → IgG4 production	High Quality	Remission duration: 8–28 months	Salvage for multidrug-refractory pemphigus
Bullous Pemphigoid	IL-5/IL-17 → JAK/STAT → Eosinophil activation	High Quality	BP lesions achieved complete remission	Experimental for steroid-resistant cases
Chronic Spontaneous Urticaria	OSMR → JAK2/STAT3 → Eosinophil recruitment	High Quality	UAS7 = 0: 42.9%–83.3%	Future third-line option

Critically, for diseases solely supported by case-level evidence, specifically pemphigus, and chronic spontaneous urticaria—the therapeutic positioning requires rigorous refinement. These agents must be expressly reserved as empirical salvage therapy for rigorously defined refractory cases unresponsive to conventional and biologic agents. Current evidence cannot establish them as standard therapy.

### Precision therapy and safety strategy

Collectively, JAK inhibitors are shifting from rescue treatments to targeted options for immune skin diseases. Strong evidence supports their use as first-choice therapies instead of steroids in conditions like alopecia areata (≥55% achieve SALT≤20), facial vitiligo (52% achieve F-VASI75), and plaque psoriasis (39%–58% achieve PASI75). However, for diseases with mixed immune pathways including pemphigus, and bullous pemphigoid, or those with limited clinical trial data, such as juvenile dermatomyositis, and chronic spontaneous urticaria, these drugs should only be considered when standard treatments fail. To improve safety, patient selection must include disease biomarkers. For patients with dermatomyositis positive for anti-MDA5 antibodies and hyperferritinemia, JAK inhibitors, while carrying infection risks demanding intensive monitoring, demonstrate lifesaving potential. Conversely in elderly or cardiovascular-compromised populations, prioritizing selective agents such as the TYK2 inhibitor envudeucitinib mitigates thrombosis-associated risks.

### Clinical practice integration

Clinical recommendations are hierarchically structured according to the GRADE framework, where high-quality evidence from randomized controlled trials (Level 1) commands primary weighting in therapeutic decision-making. For vitiligo, psoriasis, and bullous pemphigoid, multiple Phase 3 trials substantiate JAKi as first-line steroid-sparing alternatives. Conversely, conclusions for pemphigus, and chronic spontaneous urticaria rely exclusively on Level 4–5 evidence, which cannot support definitive clinical guidance due to inherent susceptibility to bias. Consequently, therapeutic proposals for these conditions are explicitly restricted to exploratory salvage scenarios in refractory disease, pending validation through controlled trials. This stratification prevents undue influence from low-certainty evidence while highlighting critical evidence gaps for future investigation.

### Safety profiles and risk mitigation

Although JAKi demonstrate transformative efficacy across diverse dermatoses, their safety profiles require vigilant risk–benefit evaluations. Class-wide adverse events include increased susceptibility to infections (e.g., herpes zoster, upper respiratory tract infections), hematologic abnormalities (e.g., anemia, neutropenia), and dyslipidemia, attributable to broad immunomodulation (113). Drug-specific risks also exist: baricitinib carries FDA boxed warnings for thrombosis, malignancy, and major adverse cardiovascular events in high-risk populations, while tofacitinib associates with elevated creatine phosphokinase (114, 115).

Risk mitigation strategies include pretreatment serologic screening (HBV, HCV, TB), complete blood cell count, lipid panel monitoring, and vaccination against herpes zoster. Crucially, severe adverse events incidence often aligns with underlying disease severity, implicating comorbid states as risk amplifiers. Despite these concerns, JAKi exhibit favorable safety over long-term glucocorticoids or non-selective immunosuppressants, positioning them as viable steroid-sparing agents with proactive surveillance.

While prior syntheses, such as Miot et al., have comprehensively examined JAKi in several dermatologic conditions (e.g., AA, vitiligo, psoriasis, DM), this work constitutes the first comprehensive synthesis encompassing both established applications (alopecia/psoriasis) and emerging frontiers—particularly autoimmune blistering diseases (pemphigus, BP) and CSU. Despite promising outcomes, current limitations include sparse randomized data for rare dermatoses and unresolved thrombosis risks in susceptible populations. Future randomized trials must validate JAKi against biologics in high-burden, steroid-dependent conditions while exploring subtype-specific safety profiles. Particularly for conditions with evidence restricted to case series, clinical implementation must require verification through controlled trials and post-marketing monitoring. To contextualize the risk-benefit profiles discussed above, the class-wide safety data of JAK inhibitors are comparatively synthesized in Table 10.

**Table 10:** Class-wide safety profile of JAK inhibitors in dermatology.

Safety parameter	Tofacitinib	Baricitinib	Upadacitinib	Ritlecitinib	Ruxolitinib
Infections	Very common	Very common	Common	Very common	Very common
Thrombosis	Uncommon	Uncommon	Uncommon	Uncommon	Uncommon
Cardiovascular	Uncommon	Uncommon	Uncommon	Uncommon	Uncommon
Malignancy	Uncommon	Uncommon	Uncommon	Uncommon	Uncommon
Lab Monitoring	CBC/LFT/Lipids/Cr	CBC/LFT/Lipids/CPK	CBC/LFT/Lipids/CPK	CBC/LFT/Lipids/CPK	CBC/LFT/Lipids
Vaccination	Avoid	Not recommended administration of live or live attenuated vaccines	Not recommended	Aviod administration of live attenuated vaccines	Not specified in the labeling

Very common: >10%; Common: 1%–10%; Uncommon: <1%.

CBC, blood cell count; LFT, liver function test; Cr, creatinine; CPK, creatinine phosphokinase.

### Future directions and knowledge gaps

Despite promising efficacy, the therapeutic expansion of JAKi in autoimmune dermatoses requires resolution of critical knowledge gaps through targeted research. Validating endotype-selective predictors underpins precision applications of JAK inhibitors. Disease-associated autoantibodies, such as anti-MDA5 in dermatomyositis, and serum CXCL10 levels in vitiligo could guide patient stratification (77, 116). Meanwhile, prospective trials should validate transcriptional biomarkers to optimize patient selection and mitigate safety risks in vulnerable subgroups.

Combination therapies require systematic investigation. For conditions with suboptimal monotherapy responses, such as non-segmental vitiligo or palmoplantar psoriasis, synergistic regimens pairing JAKi with narrowband UVB or IL-23 biologics may enhance efficacy while reducing dosage-dependent toxicities (117). Furthermore, decision between pan-JAKi and isoform-specific JAKi need phenotype-directed evaluation. For instance, selective TYK2 inhibitor deucravacitinib demonstrates superior cardiovascular safety over pan-JAKi in psoriasis, whereas diseases with polymodal cytokine activation like BP may benefit from broader JAK1/JAK2 blockade despite theoretical thrombosis risks (88, 118).

Finally, next-generation agents in clinical pipelines demand attention. Dual TYK2/JAK1 inhibitor brepocitinib exhibits enhanced kinase specificity and reduced off-target effects in early-phase trials (119). Meanwhile, next-generation JAKi like envudeucitinib, a highly selective allosteric TYK2 inhibitor demonstrating sustained efficacy and favorable 52-week safety in a phase 2 plaque psoriasis trials, could fundamentally expand the therapeutic ceiling for systemic JAK inhibition while mitigating class-wide safety liabilities (42). China-specific priorities include expediting phase IV real-world studies for domestically approved JAKi and establishing national registries to monitor long-term safety in comorbid populations. Addressing these priorities will establish JAKi as precision therapeutics beyond current steroid-dependent paradigms.
