# Supplementary material for: Therapeutic expansion of JAK inhibitors in Chinese dermatological practice: beyond atopic dermatitis to novel autoimmune frontiers
Source: Front Med (Lausanne). 2026 Aug 26;13:1752145. doi: 10.3389/fmed.2026.1752145 (PMC13558794; doi:10.3389/fmed.2026.1752145)
Supplement: Supplementary file 1 [file Table1.docx]

Table S1. Summary of Evidence Levels and Quality Assessment.

| Study Design | Evidence Level | Quality Assessment | Assessment Tool | Reference |
| --- | --- | --- | --- | --- |
| Randomized Controlled Trial (RCT) | Level 1 | High Quality | Cochrane RoB 2.0 | 57, 58, 59, 69, 70, 71, 103, 105, 110 |
| Integrated Analysis / Cohort Study | Level 2 | High Quality | Newcastle-Ottawa Scale (NOS) | 51 |
|  |  | Moderate Quality |  | 80 |
| Single-arm Clinical Trial | Level 3 | Low Quality | MINORS Scale | 46 |
|  |  | Moderate Quality |  | 50, 78 |
|  |  | High Quality |  | 79 |
| Case Series | Level 4 | Low Quality | JBI Critical Appraisal Checklist | 47 |
|  |  | Moderate Quality |  | 81 |
|  |  | High Quality |  | 48, 109, 111 |
| Case Report | Level 5 | High Quality | JBI Critical Appraisal Checklist | 49, 92, 93, 94, 95, 96, 97, 101, 102 |
